# Spatial Sorting Drives Morphological Variation in the Invasive Bird, *Acridotheris tristis*

**DOI:** 10.1371/journal.pone.0038145

**Published:** 2012-05-31

**Authors:** Cécile Berthouly-Salazar, Berndt J. van Rensburg, Johannes J. Le Roux, Bettine J. van Vuuren, Cang Hui

**Affiliations:** 1 Centre for Invasion Biology, Department of Botany & Zoology, University of Stellenbosch, Stellenbosch, South Africa; 2 School of Biological Science, University of Queensland, Queensland, Australia; 3 Centre for Invasion Biology, Department of Zoology & Entomology, University of Pretoria, Pretoria, South Africa; 4 Centre for Invasion Biology, Department of Zoology, University of Johannesburg, Johannesburg, South Africa; University of Otago, New Zealand

## Abstract

The speed of range expansion in many invasive species is often accelerating because individuals with stronger dispersal abilities are more likely to be found at the range front. This ‘spatial sorting’ of strong dispersers will drive the acceleration of range expansion. In this study, we test whether the process of spatial sorting is at work in an invasive bird population (Common myna, *Acridotheris tristis*) in South Africa. Specifically, we sampled individuals across its invasive range and compared morphometric measurements relevant and non-relevant to the dispersal ability. Besides testing for signals of spatial sorting, we further examined the effect of environmental factors on morphological variations. Our results showed that dispersal-relevant traits are significantly correlated with distance from the range core, with strong sexual dimorphism, indicative of sex-biased dispersal. Morphological variations were significant in wing and head traits of females, suggesting females as the primary dispersing sex. In contrast, traits not related to dispersal such as those associated with foraging showed no signs of spatial sorting but were significantly affected by environmental variables such as the vegetation and the intensity of urbanisation. When taken together, our results support the role of spatial sorting in facilitating the expansion of Common myna in South Africa despite its low propensity to disperse in the native range.

## Introduction

Ecologists often assume that species distributions are at their equilibriums ([1] but see [2]) and treat species' geographical ranges as though they are static features [3]. However, changing environmental conditions can profoundly affect many aspects of species' geographic ranges and thus lead to dynamic distribution patterns [4]–[5]. It is increasingly recognized that populations can undergo rapid range shifts [2]–[3], [6]–[9] and therefore dispersal ability at the edge of a species' geographic range becomes an important survival trait, particularly in the face of the negative effects of climate change [10]–[11]. In this regard, alien invasive species render excellent study systems to elucidate the evolutionary dynamics of dispersal traits in novel environments as they are often introduced to areas far removed from their historical native ranges and experience rapid range expansion.

Dispersal ability and survival in novel environments are paramount in shaping distribution ranges [12]–[13] and, not surprisingly, the evolution of dispersal in expanding populations has received much attention recently. For example, by simulating an expanding (invasive) population, Travis & Dytham [14] showed that individuals with a higher propensity to disperse were favoured at expanding edges of the distribution, resulting in increased dispersal rates at the range front. Phillips et al. [15] extended this notion by demonstrating that the dispersal kernel becomes less kurtotic and more skewed for individuals at the invading front during range expansion (invasion) events.

One potential mechanism by which dispersal is enhanced during range expansion has been termed spatial sorting, also known as spatial assortment by dispersal ability [16]–[17]. Spatial sorting can affect dispersal-relevant morphological traits such as wing size [18]–[20], leg size [21] or foot size [22] so that morphological variants associated with increased dispersal ability increases along the direction of range expansion.

Similarly, spatial sorting may also occur for cognitive-relevant traits that increase survival in novel environments. For instance, large brain size is typically associated with enhanced cognitive ability and has been linked to the establishment success and invasiveness in birds (see e.g. [23]–[24]). This is also true for behavioural traits such as aggression. For example, Duckworth [25] showed that more aggressive western bluebird individuals are more successful at expanding margins as they are more likely to occupy new habitats.

Evidently, spatial sorting is most relevant to traits that affect dispersal and fitness in novel environments. Once established, forces other than spatial sorting, such as local adaptation, competition and sexual selection [26]–[27], may act more intensely on other traits such as foraging traits (e.g. bill shape) that are mainly driven by available food resources [28]–[29]. It is therefore expected that traits associated with foraging to be not strongly affected by spatial sorting but, rather, by the characteristics of local environments. Thus, while spatial sorting will enhance dispersal-relevant traits towards a species' range margin, variation in traits such as foraging abilities will be more affected by local environmental conditions.

It is possible to determine which processes shape morphological shifts by examining trait variation across entire expanding ranges of a given species. To this end, we studied morphological variations in a highly invasive bird species, the Common myna (*Acridotheris tristis*), across its entire distribution range in South Africa. This species was accidentally introduced to Durban on the Indian Ocean coast of South Africa in 1902, followed by a second introduction (putatively independent) inland in Johannesburg (557 km west from Durban) in 1938 [30]. The Common myna has a relatively low dispersal rate compared to other bird species (dispersal distances <16 km; [31]–[32]) and is currently continually expanding its southern African range [33]. Specifically, we aim to determine whether any relationships exist between traits that are related to dispersal or survival in novel environments and the distance from the range core (i.e. the site of introduction). It is also expected that traits that are non-relevant to dispersal or survival in novel environments should not display similar relationships.

## Materials and Methods

### Morphological measurements

**Figure 1:**
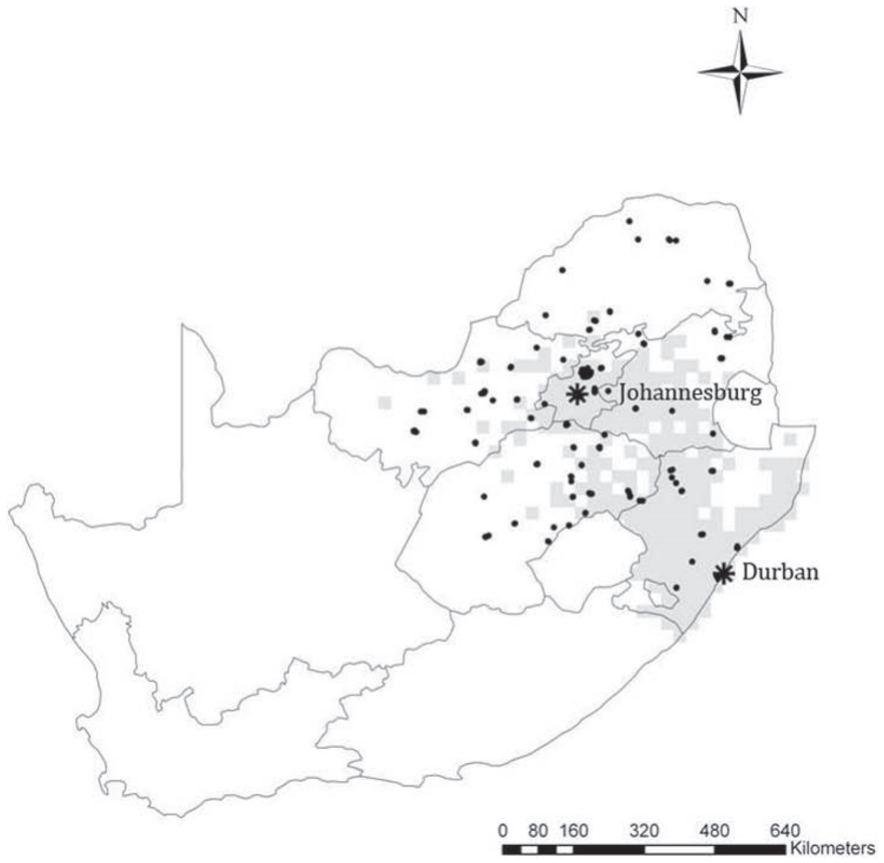
A distribution map of the Common myna (*Acridotheres tristis*) in South Africa (grey cells) based on data from [**61**]. Black dots indicate sampling sites, and the asterisks indicate the introduction sites of Durban and Johannesburg.

Common myna (*Acridotheres tristis*) specimens were collected from 58 sampling sites spanning the current non-native range across South Africa (Fig. 1). The protocol for this study was approved by the animal care committees of Pretoria University (No EC010-10). All birds used in the study were invasive species shot by hunters. Birds were sampled from public sites. Collections were carried out prior to the breeding season (June–August 2010) to ensure that female weights were not biased due to reproductive function. Carcasses were frozen at −20°C until further use. Morphological measurements were captured by one investigator (CBS) for consistency. A total of 389 adult birds were weighed (0.001 g precision) and dissected to determine the sex (217 males and 172 females). A 0.1 mm-unit dial calliper was used for all morphological measurements unless otherwise stated. Seven measurements were taken namely (a) wing length (from the carpal joint to the tip of the longest primary), (b) tarsus length (from the tibio-tarsus joint to the distal end of the tarso-metatarsus), (c) bill length (from the bill tip to the skull at nasal-frontal hinge), (d) bill depth (at the proximate edge of the nostrils), (e) bill width (at the proximate edge of the nostrils), (f) head length (from the tip of bill to the back of head, minus bill length), and (g) tail length (from the uropygial gland to the tip of the longest rectrix using a 1 mm-unit flat ruler).

To correct for size, we performed a principal component analyses (PCA) from which PC1 and PC2 can be interpreted as the size and shape variables respectively [34]. All seven morphological measurements were then separately regressed against PC1 (i.e. size) as the sole explanatory variable in a general linear model. To avoid statistical dependency between the dependent variable and PC1, we ran separate PCAs with the dependent variable removed. Significant residuals from this general linear model would indicate a deviation from the expected model and therefore significant morphological variations. These residuals were then used in all subsequent analyses. In addition, we also calculated four morphological ratios, namely (a) bill ratio (BR: bill length-to-width), (b) head ratio (HR: head-to-body length), (c) tarsus ratio (TR: tarsus-to-body length) and (d) wing-to-tail ratio (WTR), as well as wing load (the residual from the regression of log_10_[wing length^2^] by log_10_[body weight], [35]). In total, 14 morphological measurements were included in our analyses (Table 1; see also Table S1 and Table S2).

**Table 1:** Morphological traits (mean ± s.d.) and ratios of Common myna (*Acridotheres tristis*) in South Africa.

Traits	Male	Female
	(n = 217)	(n = 172)
Bill depth (mm)	8.2±0.4	7.9±0.4
Bill length (mm)	19.2±0.9	18.7±1.0
Bill width (mm)-^NS^	7.8±0.5	7.5±0.5
Head length (mm)	35.2±0.9	34.4±1.0
Tail length (mm)-^NS^	92.1±5.5	86.9±5.6
Tarsus length (mm)	38.1±1.5	36.9±1.3
Wing length (mm)	144.5±4.0	139.1±3.7
Weight (g)	124.7±11.0	113.4±8.9
WTR (wing-to-tail ratio)	0.64±0.04	0.62±0.04
BR (bill length-to width ratio)	2.50±0.19	2.52±0.20
HR (head-to-body length ratio)	3.54±0.29	3.30±0.25
TR (tarsus-to-body length ratio)	3.27±0.27	3.08±0.21
Wing loadings	0.01±0.02	0.01±0.02

NS: traits displaying non-significant dimorphism between sexes.

### Environmental variables

We compiled a set of 11 environmental variables, representing variation in climate, habitat and human impact, across all sampling sites (Table 2). Four bioclimatic variables namely mean winter precipitation, mean summer precipitation, mean summer maximum temperature and mean winter minimum temperature were obtained from the WorldClim database [36]. Habitat variables include altitude, the normalised difference vegetation index (NDVI, January mean from 1982 to 1999, [37]) and the core distance measured as distance from the sampling sites to the possible centre of introduction (Johannesburg and Durban). It is worth noting that distance from the sampling sites to Johannesburg is not inversely related to the distance from the same sampling sites to Durban. Instead, both reflect core distances of each introduction event, i.e. Johannesburg and Durban, and can be used as explanatory variables in a multivariate analysis. We performed a PCA on five transformed land covers variables, namely, cultivated land, degraded land, tree plantation, irrigation and urban build-up [37] calculated at the quarter degree resolution derived from 1994 and 1995 Landsat TM satellite images [38]. The two first axes of the PCA, named Urban 1 and 2, were used as variables indicative of the Human impact.

**Table 2:** Summary of environmental variables range in sampling sites.

Categories	Environmental variables	Minimum value	Maximum value
Habitat	Distance from Johannesburg (km)	4.5	497.5
	Distance from Durban (km)	3.6	818.9
	Normalised Difference of Vegetation Index	0.21	0.48
	Altitude (m)	13	1797
Climate	Winter Minimum temperature (°C)	−3.2	11.6
(means)	Summer Maximum temperature (°C)	23.8	31.7
	Summer precipitation (mm)	66	156
	Winter precipitation (mm)	2	35
Human	National road distance (km)	0.009	154.6
impact	Cultivated area (% in QDS)	0	90.7
	Degraded land (% in QDS)	0	26.1
	Tree plantation (% in QDS)	0	60.2
	Irrigation area (% in QDS)	0	32.3
	Urban build-up (% in QDS)	0	85.7

NDVI: normalized vegetation index; QDS: quarter degree cell.

### Statistical analyses

#### Multi-scale pattern analyses

To investigate the spatial structure in our data, we used a PCA in which independent variables were regressed with predictors of spatial connectivity. This approach has been termed multi-scale pattern analysis (MSPA) [39] and is implemented in the package *adegenet* from the R statistical environment [40]. Specifically, we used Moran's eigenvector maps (MEMs), derived from Moran's index for spatial autocorrelation as spatial predictors [41]. MEMs have been shown to be good spatial predictors ([39] and reference therein). MEMs are named in a decreasing order, where MEM_1_ describes the spatial connectivity at the broadest spatial scale, with subsequent MEMs, describing spatial connectivity at reducing scales and the last MEM at the finest local spatial scale. The determination coefficient, R^2^, obtained from the MSPA was used to quantify the strength of association between the independent variables and MEMs [41]. Signals of spatial structure at particular scales in morphological measurements and environmental variables were detected for each sex separately (i.e. a total of four MSPAs). We then applied a redundancy analysis [42] to the MSPA for explaining the morphological variables by environmental variables at all spatial scales. This enabled us to combine spatial predictors and environmental variables simultaneously to describe the variation in morphological traits. A schematic description of the full procedure is summarised in Figure S1.

#### Linear mixed model

While the previous analysis was aimed at highlighting correlations between morphological traits and environmental variables across spatial scales, it will not provide the statistical significance of these interactions. Therefore, we performed a linear mixed model (LMM) for each morphological trait with the sampling site as a random factor and environmental variables as explanatory variables. The best model was identified by the Akaike information criterion (AIC, [43]) and the significance of each covariate tested using a Monte Carlo approach retaining only the significant covariates. When more than one variable was retained in the model, the potential collinearity between explanatory variables was also checked. If two explanatory variables exhibited a notable correlation (R^2^>0.1), the model was re-run for each variable separately to check whether significance was retained for each variable separately. An explanatory variable was removed from the final model if no significant effect was found. If both variables showed significant effects when run separately, both were retained in the model. We used a sequential regression to prioritise each explanatory variable by its significance and thus reduced the collinearity between covariates [44]. The LMM was first run for the entire dataset (females and males combined) and then retained models were run for each sex separately. This allowed us to examine whether environmental determinants, identified in the final model, are sex-specific.

## Results

### Morphometric MSPA

The eigenvalues of the multi-scale pattern analysis (MSPA) for the morphological traits indicated that the first two principal component axes explained 40% and 42% of the variation in females and males respectively. For Moran's eigenvector maps (MEMs), we focused only on those variables that accounted for more than 3% of the morphological variation (see Fig. S1). Although this value is somewhat arbitrary, it nonetheless represents the point after which there was a strong decrease in the percentage of variation explained. Consequently, we identified five MEMs for females (MEM_1, 2, 6, 29 and 37_) representing broad-scale structures and two MEMs for males (MEM_1_ at the broadest spatial scale and MEM_209_ at a local scale). Spatial connectivity among samples failed to explain the observed variation in morphological traits as R^2^ values were lower than 0.1 in both sexes. For females, two morphological traits (bill width and tail length) and bill ratio (BR) displayed the strongest, albeit still weak, spatial structures with about 13 to 17% of their variation explained by the two first principal components of the MSPA. For males, a maximum of only 11% of variation explained for two traits (tail length and WTR).

### Environmental MSPA

The spatial structure was strong in environmental variables and the eigenvalues of the MSPA demonstrated that the first two principal component axes explained more than 80% of the variation. Environmental spatial structure was consistently detected at broader scales (MEM_1–4_). The broadest scale MEM_1_ clearly resonated with the distance from Johannesburg (R^2^>0.4). The second and third MEMs (i.e. MEM_2_ and MEM_3_) were found mainly related to environmental factors that describe habitat quality, such as NDVI, altitude, temperature and precipitation (0.2<R^2^<0.6).

### Spatial and environmental MSPA

**Figure 2:**
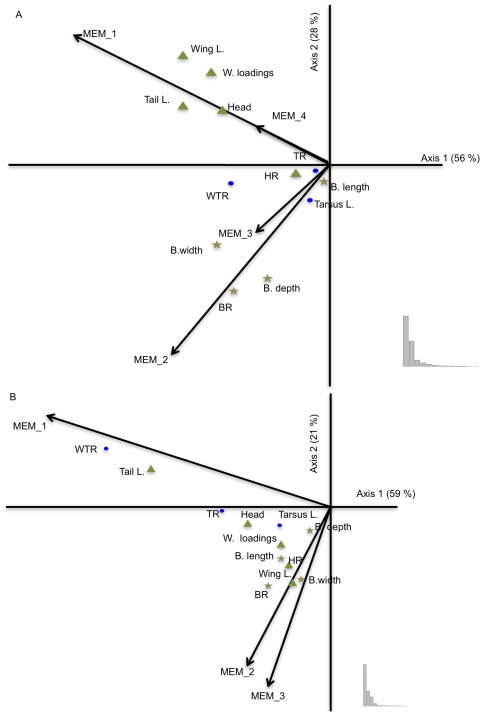
Results from the environmental and morphological analysis using the MSPA redundancy analysis for females (A) and for males (B). Eigenvalues are shown as insets. Triangles indicate traits related to flight, circles indicate traits related with tarsus, and stars indicate traits related with bill.

The MSPA redundancy analysis regressing morphological traits against environmental variables revealed some clear structure at different spatial scales (Fig. 2). For females, the first-group spatial predictors (MEM_1_ and MEM_4_) were related to traits influencing dispersal and cognitive capacity (wing length, wing loadings, tail length and head size) and explained 34 to 53% of the morphological variation (Fig. 2A). The second-group spatial predictors (MEM_2_ and MEM_3_) were largely orthogonal to the first-group spatial predictors and concerned mainly traits related to foraging activities (bill size, bill ratio, bill depth and bill width), with 38 to 47.5% of the morphological variation explained (Fig. 2A). Notably, the spatial predictors MEM_1_ and MEM_4_ were also associated with the distance from Johannesburg whilst the spatial predictors (MEM_2_ and MEM_3_) were related to the distance from Durban and other environmental factors of habitat quality. For males, the spatial predictor MEM_1_ explained 58% of the variation in tail length and 74% of the variation in wing-tail ratio (WTR), whilst the second-group spatial predictors (MEM_2_ and MEM_3_) were weakly linked to nearly all the other traits, explaining from 7% of the variation in tail length to 45% of the variation in wing length, with no preferential traits highlighted (Fig. 2B).

### Linear regression

When both sexes were included, the LMM analysis revealed significant sexual dimorphism in the Common myna, with females being smaller for all traits except for tail length, shape (PC2), bill ratio (BR) and bill width (Table 3). Sex explained 70 to 97% of the variation in morphological traits. Dispersal traits (wing length, wing loading and tail length) increased with altitude and the distance to Johannesburg, whereas birds collected further away from Johannesburg had bigger heads.

**Table 3:** Summary of environmental variables with significant effect on traits measured using linear regression models.

	Female Sex	Winter precipitation	Temperature maximum	NDVI	Altitude	Distance from Johannesburg	National road distance	Urban 2
Bill length	-							+|m(+)
Bill width			+|m(+)	+|f(+)				
Bill depth	-							−|m(−)
Head	-					+|f(+)		
Tail L.					+|m(+)	+|m(+)		
Tarsus L.	-	−|m(−)			−|m(−)|f(−)			
Wing length	-				+|m(+)|f(+)	+|f(+)		
Wing loading	-				+|m(+)|f(+)	+|f(+)		
HR	-			−|m(−)|f(−)				
TR	-			−|m(−)	−|m(−)			
BR	-			−|m(−)|f(−)	+|m(+)|f(+)			
WTR	-	+|m(+)						
Size	-						+|m(+)	
Shape	-			+|f(m)	+|f(+)			

Signs indicate positive or negative effects. Plain cells indicate that only interactions of the two factors have an effect. Each effect has been tested for females (f) and males (m) separately. *+|m(+) : indicates a positive effect when both sexes combined (+|), when sexes were analysed separately only a positive effect was found in males (m(+)).*

**Figure 3:**
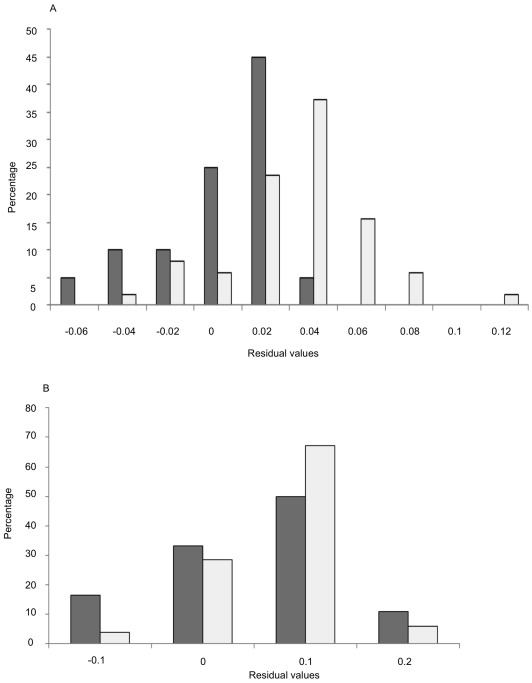
Distribution histograms for traits residuals of female head size (A) and female bill width (B). Solid bars indicate individuals at the range core (within the 50 km radius of Johannesburg), and open bars indicate individuals at the range margin (beyond 300 km but excluding individuals from the Durban population).

When the LMM was run for males and females separately, some of the environmental factors that have significant effects on morphological traits were found to be sex-specific (Table 3). For females, wing length and wing loading were influenced by altitude and the distance to Johannesburg, whereas only altitude had a significant effect on these traits in males. Similarly, only female head size increased with the distance to Johannesburg, and only the tail of male birds increased with both altitude and the distance to Johannesburg. Female tarsus was found shorter at the high altitude, whereas male tarsus dwindled with both altitude and winter precipitation. The environmental variable Urban 2, corresponding to degraded areas and commercial tree plantations, had a significant impact on male birds to have elongated and flattened bills (i.e. increasing bill length but reducing bill depth), whereas the bill width in male birds increased with the maximum temperature. The vegetation index NDVI was found mainly affecting head and bill ratios for both sexes. Value distributions of traits that have been subjected to spatial sorting are expected to shift between individuals at the core and at the edge of the distribution. We plotted value distributions for two traits that were only influenced by one factor to ensure the observation of only one effect. Head size in females (cognitive traits influence by the distance to Johannesburg) showed a clear shift due to spatial sorting between core and marginal individuals (Fig. 3A), whereas bill width in females (foraging traits) showed no obvious shift and thus a weak effect of spatial sorting (Fig. 3B).

## Discussion

Spatial sorting theory predicts that dispersal ability should be enhanced at the range front of an expanding population into novel habitats. In other words, a significant positive correlation between dispersal ability and distance from the core of the distribution should be observed. This was supported by our results where a significant correlation was found between one of the core distances (Johannesburg) and traits related to dispersal ability (i.e. flying). The spatial assortment for stronger dispersers at the expanding edge creates a positive feedback that can accelerate the speed of range expansions. Such accelerating range expansion has been commonly identified in many highly invasive species, such as bush crickets [45] and cane toads [21]. Evidently, ever-increasing dispersal ability at the range margin, manifested through dispersal and cognitive traits, could play an important role in range dynamics and invasion success [21].

If sex-biased dispersal occurs in a particular species, one would expect a stronger effect from spatial sorting on one of the sexes. This is indeed the case for Common mynas in South Africa. Dispersal-relevant traits in females, specifically wing measurements (and also head size), showed a strong correlation with the distance to one of the introduction sites (Johannesburg) while no such a correlation was detected for males. Although no studies on the dispersal of Common mynas are available, the species does show signs of female-biased dispersal, consistent with Greenwood's [46]–[47] proposal that female-biased dispersal and male-biased philopatry are common in birds. Specifically, dispersal strategies are often linked to mating systems, resulting in resource defence in monogamy (typical in birds) where males take the lead role in acquisition and defence of resources and receive considerable benefits by remaining philopatric. As males are often more subjected to predation and aggression [48]–[49], aggression-related traits (such as tail length) may be more affected in males and therefore could explain the high morphological variation in tails for male Common mynas. In addition, sex biased dispersal could potentially lead to different sex ratios in expanding populations (e.g. a slightly lower, yet insignificant, sex ratio ( = 0.45) was found for birds within 250 km radius to Johannesburg than beyond (sex ratio = 0.49)).

An increase in cognitive ability would strongly favour individuals by allowing them to better cope under novel environmental conditions [23]–[24], [25], [50]. Head size is a qualitative proxy for brain size [24] and, subsequently, cognitive abilities [23]. For instance, increased head size has been linked to increased offspring defence and higher success at expanding range edges [25]. Head size in female Common mynas, the dispersing sex, increased significantly with distance away from one of the points of introduction (Johannesburg).

Why a significant correlation was found between many morphological traits and the distance to Johannesburg but not to Durban remains unknown. Two possible explanations warrant further investigation. First, dispersal-related traits often become homogenized once the range expansion stops [15] so that while the spatial sorting influences morphological variation in expanding populations, its effect will be diluted once populations reach their equilibriums. Specifically, when populations no longer expand, the backward migration of individuals from the margins to the core will homogenize morphological variations across the distribution range. Since the introduction to Durban predates the introduction to Johannesburg by nearly thirty years [33], it may be that the Durban expansion has potentially filled up most suitable habitats and reached the distributional equilibrium. Second, distinct environmental characteristics of these two introduction points could have differentially influenced populations' expansion. Johannesburg is located within the grassland biome of South Africa, bordering the savannah biome [51], whereas Durban is located within a subtropical thicket that extends along the east coast of the country. While the open grassland savannah may be more conducive to dispersal, the thicket and coastal forests surrounding Durban but also the Drakensberg mountain ridge seems impenetrable and may have contributed to prevent high levels of dispersal from this coastal introduction point.

Spatial sorting favours dispersal-relevant traits and those that provide a benefit at the range front during range expansion. Traits that do not provide any benefits in novel environments should therefore not show strong spatial sorting effect. Here we found that foraging traits in Common myna show no significant correlation with distance from the core. Conversely, factors of habitat quality could affect these non-dispersal-related traits. This is exactly what we found between the morphological variation in foraging-relevant traits and environmental variables (e.g. NDVI and urbanization). Specifically, urbanization can modify the quality and type of food resources and therefore influence bill shape (bill length and depth), consistent with previous studies [28]–[29]. As an indicator of primary productivity (and thus the habitat quality and food resources), NDVI was found to significantly influence the head ratio (HR) and bill ratio (BR) in both sexes. Besides food resources, other environmental factors are also expected to influence morphology and we found winter precipitation to negatively influence tarsus length. Similarly altitude appears to affect tarsus length negatively but wing and tail length positively. The latter findings are consistent with the evidence from Red-billed chough (*Pyrrhocorax phyrrhocorax*) and Alpine chough (*Pyrrhocorax graculus*) [52].

Spatial sorting has been one of the main hypotheses put forward to explain the increase in dispersal abilities in alien invasive species during range expansion. Spatial sorting allows dispersal-enhancing traits to accumulate at the expanding range front in only a few generations through phenotypic plasticity. Moreover, since dispersal can affect the processes of intraspecific competition and kin (inbreeding) avoidance, it is also under classical natural selection [14]. High dispersal rates can enable individuals to reduce the intensity of kin competition [53] and therefore could have an evolutionary advantage at the range front [54]–[55]. However, high dispersal rates can also act to constrain adaptation at the periphery and slow down range expansion [56], [57]. The potential trade-off between dispersal and reproduction can further complicate any simple deductions from classical natural selection [58]. Therefore, the roles of spatial sorting (which does not rely upon fitness differentials) vs. natural selection (which assumes fitness differentials) during range expansion, while not mutually exclusive, remains a matter of debate [17], [59]–[60]. Spatial sorting generally acts at a faster pace than natural selection and thus suffices to enhance dispersal traits at the range front of a younger population; however, genetic analyses and common garden experiments are needed to quantify the relative contribution from each of these processes during range expansion.

In conclusion, we found that Common mynas in South Africa seemed to be subjected to spatial sorting and consequently dispersal-relevant traits showed a shift towards higher dispersal ability at the range of the species' current distribution (e.g., see [17]). Our findings confirm that this invasive species has enhanced its dispersal ability during the range expansion under novel environmental conditions despite being a relatively poor disperser in its native area. This has important implications for invasion biology in general as life-history traits from a species' native range are often used to inform ecologists about the potential to disperse and spread in non-native ranges [27].

## Supporting Information

Figure S1 **Schematic analytical procedure.**
(DOC)

Table S1 **Summary of morphological measurements and ecological variables used.**
(DOC)

Table S2 **Mean and standard deviation of morphometric measurements per locality per sex.**
(XLSX)

## References

[1] Pearson RG, Dawson TP (2003). Predicting the impacts of climate change on the distribution of species: are bioclimate envelope models useful?. Global Ecol Biogeogr.

[2] Václavýk T, Meentemeyer RK (2012). Equilibrium or not? Modelling potential distribution of invasive species in different stages of invasion.. Divers Distrib.

[3] Channell R, Lomolino MV (2000). Trajectories to extension: spatial dynamics of the contraction of geographical ranges.. J Biogeogr.

[4] Svenning J-C, Skov F (2004). Limited filling of the potential range in European tree species.. Ecol Lett.

[5] Araújo MB, Pearson RG (2005). Equilibrium of species' distributions with climate.. Ecography.

[6] Brown JH, Stevens GC, Kaufman DM (1996). The geographic range: size shape boundaries and internal structures.. Annu Rev Ecol Syst.

[7] Davis MB, Shaw RG (2001). Range shifts and adaptive responses to quaternary climate change.. Science.

[8] Holt RD (2003). On the evolutionary ecology of species' ranges.. Evol Ecol Res.

[9] Sekerciolu CH, Loarie SR, Brenes FO, Ehrlich PR, Daily GC (2007). Persistence of forest birds in the Costa Rican Agricultural countryside.. Cons Biol.

[10] Peterson AT (2003). Projected climate change effects on rocky mountain and great plains birds: generalities of biodiversity consequences.. Global Change Biol.

[11] Malcolm JR, Liu C, Neilson RP, Hansen L, Hannah L (2006). Global warming and extinctions of endemic species from biodiversity hotspots.. Cons Biol.

[12] Bridles JR, Vines TH (2007). Limits to evolution at range margins: when and why does adaptation fail?. Trends Ecol Evol.

[13] Gaston KJ (2009). Geographic range limits of species.. Proc R Soc B.

[14] Travis JMJ, Dytham C (2002). Dispersal evolution during invasions.. Evol Ecol Res.

[15] Phillips BL, Brown GP, Travis JMJ, Shine R (2008). Reid's paradox revisited: the evolution of dispersal kernels during range expansion.. Am Nat.

[16] Phillips BL, Brown GP, Shine R (2010). Life-history evolution in range-shifting populations.. Ecology.

[17] Shine R, Brown GP, Phillips BL (2011). An evolutionary process that assembles phenotypes trough space rather than trough time.. Proc Natl Acad Sci U S A.

[18] Leisler B, Winkler H, Berthold P, Gwinner E, Sonnenschein E (2003). Morphological consequences of migration.. Avian Migration.

[19] Dawideit BA, Phillimore AB, Laube I, Leisler B, Böhning-Gaese K (2009). Ecomorphological predictors of natal dispersal distances in birds.. J Anim Ecol.

[20] Baldwin MW, Winkler H, Organ CL, Helm B (2010). Wing pointedness associated with migratory distance in common-garden and comparative studies of stonechats Saxicola torquata.. J Evol Biol.

[21] Phillips BL, Brown GP, Webb JK, Shine R (2006). Invasion and the evolution of speed in toads.. Nature.

[22] Forsman A, Merilä J, Ebenhard T (2011). Phenotypic evolution of dispersal-enhancing traits in insular voles.. Proc R Soc B.

[23] Sol D, Duncan RP, Blackburn TM, Cassey P, Lefebvre L (2005). Big brains enhanced cognition and response of birds to novel environments.. Proc Natl Acad Sci U S A.

[24] Møller AP (2010). Brain size head size and behaviour of a passerine bird.. J Evol Biol.

[25] Duckworth RA (2008). Adaptive dispersal strategies and the dynamics of a range expansion.. Am Nat.

[26] Lockwood JL, Hoopes MF, Marchetti MP (2007). Invasion ecology..

[27] Blackburn TM, Lockwood JL, Cassey P (2009). Avian Invasions The ecology and evolution of exotic birds..

[28] Radford AN, Du Plessis MA (2003). Bill dimorphism and foraging niche partitioning in the green woodhoope.. J Anim Ecol.

[29] Grant PR, Grant BR (2006). Evolution of character displacement in Darwin's finches.. Science.

[30] Hockey PAR, Dean WRJ, Ryan PG, Maree S (2005). Roberts' birds of southern Africa (7th edn)..

[31] Slocum GL (1995). Could the Common Myna (*Acridotheres tristis*) be managed by habitat manipulation in the ACT?.

[32] Kang N (1992). Radio telemetry in an urban environment: a study of mynas (Acridotheres spp) in Singapore Wildlife telemetry: remote monitoring and tracking of animals..

[33] Peacock DS, van Rensburg BJ, Robertson MP (2007). The distribution and spread of the invasive alien Common myna *Acridotheres tristis* L. Aves: Sturnidae in southern Africa.. S Afr J Sci.

[34] Rising JD, Somers KM (1989). The measurement of overall body size in birds.. The Auk.

[35] Marchetti K, Price T, Richman A (1995). Correlates of wing morphology with foraging behaviour and migration distance in the genus Phylloscopus.. J Avian Biol.

[36] Hijmans RJ, Cameron SE, Parra JL, Jones PG, Jarvis A (2005). Very high resolution interpolated climate surfaces for global land areas.. Int J Climatol.

[37] Hugo S, van Rensburg BJ (2008). The maintenance of a positive spatial correlation between South African bird species richness and human population density.. Global Ecol Biogeogr.

[38] Thompson M (1996). A standard land-cover classification scheme for remote-sensing applications in South Africa.. S Afr J Sci.

[39] Jombart T, Dray S, Dufour A-B (2009). Finding essential scales of spatial variation in ecological data: a multivariate approach.. Ecography.

[40] R Core Development Team (2006). R: A language and environment for statistical computing..

[41] Dray S, Legendre P, Peresneto P (2006). Spatial modelling: a comprehensive framework for principal coordinate analysis of neighbour matrices PCNM.. Ecol Model.

[42] Rao CR (1964). The use and interpretation of principal component analysis in applied research.. Sankhyà Ser A.

[43] Akaike H (1974). A new look at the statistical model identification.. IEEE T Automat Contr.

[44] Graham MH (2003). Confronting multicollinearity in ecological multiple regression.. Ecology.

[45] Simmons AD, Thomas CD (2004). Changes in dispersal during species range expansions.. Am Nat.

[46] Greenwood PJ (1980). Mating systems phylopatry and dispersal in birds and mammals.. Anim Behav.

[47] Greenwood PJ, Harvey PH (1982). The natal and breeding dispersal of birds.. Annu Rev Ecol Syst.

[48] Hendry AP, Kelly ML, Kinnison MT, Reznick DN (2006). Parallel evolution of the sexes? Effects of predation and habitat features on the size and shape of wild guppies.. J Evol Biol.

[49] Oufiero CE, Garland T (2007). Evaluating performance costs of sexually selected traits.. Funct Ecol.

[50] Saino N, Cuervo JJ, Krivacek M, Lope FD, Møller P (1997). Experimental manipulation of tail ornament size affects the hematocrit of male barn swallows *Hirundo rustica*.. Oecologia.

[51] Mucina K, Rutheworth MC (2006). The vegetation of South Africa Lesotho and Swaziland..

[52] Laiolo P, Rolando A (2008). Ecogeographic correlates of morphometric variation in the Red-billed Chough *Pyrrhocorax pyrrhocorax* and the Alpine Chough *Pyrrhocorax graculus*.. Ibis.

[53] Phillips BL (2009). The evolution of growth rates on an expanding range edge.. Biol Lett.

[54] Higgins SI, Nathan R, Cain ML (2003). Are long-distance dispersal events in plants usually caused by non standard means of dispersal?. Ecology.

[55] Bowler DE, Benton TG (2005). Causes and consequences of animal dispersal strategies: relating individual behaviour to spatial dynamics.. Biol Rev.

[56] Kirkpatrick M, Barton NH (1997). Evolution of a species' range.. Am Nat.

[57] Sakai AK, Allendorf FW, Holt JS, Lodge DM, Molofsky J (2001). The population biology of invasive species.. Annu Rev Ecol Syst.

[58] Travis JMJ, Mustin K, Benton TG, Dytham C (2009). Accelerating invasion rates result from the evolution of density-dependant dispersal.. J Theor Biol.

[59] Shine R, Brown GP, Phillips BL (2011). Reply to Lee: Spatial sorting, assortative mating and natural selection.. Proc Natl Acad Sci USA.

[60] Lee MSY (2011). Macroevolutionary consequences of “spatial sorting”.. Proc Natl Acad Sci USA.

[61] Harrison JA, Allan DG, Underhill LG, Herremans M, Tree AJ (1997). The atlas of southern African birds..

