# Supplementary material for: Spatial Sorting Drives Morphological Variation in the Invasive Bird, Acridotheris tristis
Source: PLoS One. 2012 May 31;7(5):e38145. doi: 10.1371/journal.pone.0038145 (PMC3364963; doi:10.1371/journal.pone.0038145)
Supplement: Figure S1 — Schematic analytical procedure. (DOC) [file pone.0038145.s001.doc]

**Figure S1.** Summary of the multivariate procedures. n_i_ = number of individuals; n_s_ = number of sampling sites. For this study, n_i=_ n_s_
