# Supplementary material for: Spatial Sorting Drives Morphological Variation in the Invasive Bird, Acridotheris tristis
Source: PLoS One. 2012 May 31;7(5):e38145. doi: 10.1371/journal.pone.0038145 (PMC3364963; doi:10.1371/journal.pone.0038145)
Supplement: Table S1 — Summary of morphological measurements and ecological variables used. (DOC) [file pone.0038145.s002.doc]

Table S1. Summary of morphological measurements and ecological variables used

| **Traits** | **Adjustment** |  |
| --- | --- | --- |
| 1. Bill Depth (mm) | size-adjusted |  |
| 1. Bill Length (mm) | size-adjusted |  |
| 1. Bill Width (mm) | size-adjusted |  |
| 1. Head Length (mm) | size-adjusted |  |
| 1. Tail Length (mm) | size-adjusted |  |
| 1. Tarsus Length (mm) | size-adjusted |  |
| 1. Wing Length (mm) | size-adjusted |  |
| 1. ***Size*** | ***PC1 from 7 measurements above*** |  |
| 1. ***Shape*** | ***PC2 from 7 measurements above*** |  |
| 1. Wing-to-tail ratio (WTR) |  |  |
| 1. Bill length-to-width (BR) |  |  |
| 1. Head-to-body length (HR) |  |  |
| 1. Tarsus-to-body length (TR) |  |  |
| 1. Wing loadings |  |  |
| Weight (g) |  |  |
| **Environmental variables** | **Combined by PCA analysis** |  |
| 1. Mean Temperature minimum (C) |  |  |
| 1. Mean Temperature maximum (C) |  |  |
| 1. Mean Summer precipitation (mm) |  |  |
| 1. Mean Winter precipitation (mm) |  |  |
| 1. Altitude (m) |  |  |
| 1. Distance from Johannesburg (Km) |  |  |
| 1. Distance from Durban (Km) |  |  |
| 1. N road (Km) |  |  |
| 1. NDVI (normalized vegetation index) |  |  |
| 1. ***Urban1*** 2. ***Urban2*** | ***Cultivated area (% in QDS)*** |  |
|  | ***Degraded Land (% in QDS)*** |  |
|  | ***Trees plantations (% in QDS)*** |  |
|  | ***Irrigation areas (% in QDS)*** |  |
|  | ***Urban build up (% in QDS)*** |  |
